# Drift Reduction in Pedestrian Navigation System by Exploiting Motion Constraints and Magnetic Field

**DOI:** 10.3390/s16091455

**Published:** 2016-09-09

**Authors:** Muhammad Ilyas, Kuk Cho, Seung-Ho Baeg, Sangdeok Park

**Affiliations:** 1 Department of Robotics and Virtual Engineering, University of Science and Technology (UST), Daejon 305-333, Korea; milyasmeo@kitech.re.kr; 2 Robotics R & BD Group, Korea Institute of Industrial Technology (KITECH), Ansan 426-791, Korea; googi33@kitech.re.kr (K.C.); sdpark@kitech.re.kr (S.P.)

**Keywords:** pedestrian navigation system, drift reduction in INS, magnetic anomaly detection, multi-sensor fusion

## Abstract

Pedestrian navigation systems (PNS) using foot-mounted MEMS inertial sensors use zero-velocity updates (ZUPTs) to reduce drift in navigation solutions and estimate inertial sensor errors. However, it is well known that ZUPTs cannot reduce all errors, especially as heading error is not observable. Hence, the position estimates tend to drift and even cyclic ZUPTs are applied in updated steps of the Extended Kalman Filter (EKF). This urges the use of other motion constraints for pedestrian gait and any other valuable heading reduction information that is available. In this paper, we exploit two more motion constraints scenarios of pedestrian gait: (1) walking along straight paths; (2) standing still for a long time. It is observed that these motion constraints (called “*virtual sensor*”), though considerably reducing drift in PNS, still need an absolute heading reference. One common absolute heading estimation sensor is the magnetometer, which senses the Earth’s magnetic field and, hence, the true heading angle can be calculated. However, magnetometers are susceptible to magnetic distortions, especially in indoor environments. In this work, an algorithm, called magnetic anomaly detection (*MAD*) and compensation is designed by incorporating only healthy magnetometer data in the EKF updating step, to reduce drift in zero-velocity updated INS*.* Experiments are conducted in GPS-denied and magnetically distorted environments to validate the proposed algorithms.

## 1. Introduction

Micro-electromechanical systems (MEMS) technology that combines micro-processors with tiny mechanical sensors embedded in semiconductor chips has rationalized the concept of the “*Ubiquitous Localization*”. The core topic in ubiquitous localization is to track the position of an agent anywhere/any time i.e., indoors or outdoors. The location based services (LBS) use a variety of sensors and systems e.g., using GPS signals, RFID, WLAN/Wi-Fi, ultrasound, radio or vision technology [1,2] to track the location of a mobile agent. However, these services usually require pre-installation of localization beacons in a given environment to be used for localization. It becomes hard to install navigation sensors/system in many kinds of applications, e.g., in hostile or hazardous environments, dangerous or collapsed buildings, emergency situations, etc. Therefore, infrastructure-free (i.e., with no navigational beacons) navigation solutions are preferred over infrastructure-based navigation since they do not depend on any pre-condition [3].

Infrastructure-free localization has been widely researched during the last decades. Algorithms and systems have been proposed for personal localization based on inertial sensors only [4,5,6,7,8]. The MEMS type inertial sensors are used in two ways within PNS [9]. In the first approach, called Pedestrian Dead-Reckoning (PDR) solution, the inertial sensor (s) is mounted on any body part, other than the foot. In PDR, a constant step length is assumed on a relatively smooth surface, often usable in office-like environments. In PDR, the average step length is integrated along with orientation estimates obtained from IMU at each detected step to track the position of a mobile agent. This technique is simple and does not depend on the double integration of accelerometer signals to get position estimates. The downside of PDR is that it is applicable on relatively smooth surfaces and assumes a constant step length, however, it needs tuning for a specific user. The second approach, called the inertial navigation system (INS), is adapted from the aerospace community, in which IMU is used at a high rate for tracking position, velocity and attitude (PVA) of the platform on which it is fixed/strapped-down [10,11]. It is well known that using only IMU is subject to drift in navigation solutions, depending on the precision of the sensor used. High-end inertial sensors, e.g., using ring-laser or fiber-optic gyro, are very costly and bulky for use in PNS applications, as the main limitation in PNS is size, weight and power consumption of the sensors. To alleviate drift issues in INS, GPS is often used in outdoor navigation applications. However, from a pedestrian navigation perspective, users are not always under the open sky; hence, GPS is not a reliable aid to INS. Using other sensors makes the system more dependent, complex and costly/bulky; hence, we investigate the use of only a single IMU fitted on the foot of the agent and assume no pre-conditions for the user and the operating environment.

In this paper, we exploit the waking constraints of a user with IMU fitted on one of their feet, and apply an appropriate algorithm to reduce the drift in the INS-only solution [12]. The following motion constraints are considered in this paper:

- Stance phase i.e., conventionally used Zero-velocity update (*ZUPT*)
- Walking along straight paths i.e., Straight Trajectory Heading update (*STH*)
- Standing still for a long time i.e., Position and Attitude Lock (*PA-Lock*).

**Figure 1:**
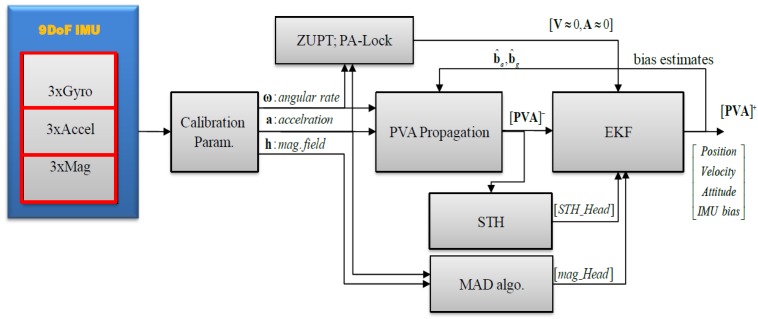
Pedestrian Navigation System (PNS) aided with motion constraints and healthy magnetic field data.

In addition to exploiting these motion constraints, we also made use of magnetometer data to reduce drift in a navigation solution. A triad of gyroscopes can be used for tracking all three angles once initialized properly, for a considerable length of time, depending on the accuracy of the sensor. However, attitude angles estimated using IMU-only tend to drift with the passage of time, if not corrected by some absolute reference. Though roll and pitch (tilt) angles are easy to correct due to gravity vector sensing by the accelerometer; correction of the absolute heading angle is more challenging for MEMS IMU. Different techniques are employed for absolute heading angle estimation, depending on the type of sensors and the accuracy requirements; among them are: Gyro compassing, Magnetometer, Sun sensor, Star trackers, Multi-antenna GPS receiver, Map matching, etc. Each technique has its own limitations and merits. 3D magnetometers can be used for absolute heading angle tracking in a magnetically clean environment [13]. However, a magnetometer does not work reliably in magnetically distorted environments. In this work, we have developed an algorithm to overcome this limitation of traditional navigation systems by employing magnetometers. However, before using magnetic field readings, we continuously checked the “*health*” of the magnetometer data using the proposed *Magnetic Anomaly Detection (MAD)* technique, determining whether the sensor output is suitable for heading calculations. A diagram illustrating the design of the PNS in this paper is shown in Figure 1 below.

One big advantage of using magnetometer data in this way in an EKF framework is that distorted magnetometer data does not disturb estimation of roll and pitch angles, in contrast to conventional approaches in AHRS design [13,14].

The layout of this paper is as follows: In Section 2, we describe the INS mechanization for foot-mounted PNS in details; Section 3 explains the INS aiding with virtual sensors (motion constraints); and, in Section 4, we present the use of magnetometer based heading calculations and developed a *MAD* algorithm; In Section 5, the design of EKF for PNS application is presented; Section 6 provides experimental work and results from the application of virtual sensors to reduce PNS position drift, and Section 7 presents *MAD* algorithm testing and application to the PNS system; Finally, Section 8 concludes this work and offers some future research suggestions.

## 2. Foot-Mounted Inertial Navigation System: Designing PNS

### 2.1. Foot-Mounted INS Mechanization

Inertial navigation system mechanization equations are a set of equations which form the basis of inertial navigation in a specified reference frame. Depending on the application, these equations take slightly different forms in different navigation frames. It is well known that IMU output is related to the navigation information (i.e., PVA) through kinematic equations, known as “*INS Mechanization*” [10,15]. The acceleration of a mobile agent in a navigation frame is expressed as follows:

(1) $${\dot{V}}^{n}=C_{b}^{n}f^{b}-\left(2\omega_{ie}^{n}+\omega_{en}^{n}\right)\times V^{n}+g^{n}$$

The first term on the R.H.S is the specific force (SF) experienced by accelerometer transformed in the navigation frame by the transformation matrix ($C_{b}^{n}$), which in turn is formed by angular rates sensed by gyroscopes. The second term is the Coriolis acceleration due to the rotation of the Earth itself and the last term is the effective gravity vector expressed in the local navigation frame. The terms $\omega_{ie}^{n}$ and $\omega_{en}^{n}$ are the Earth rotation vector and navigation frame transport rate vector, respectively, which are ignored for PNS applications. Hence, the effective acceleration for PNS application in Earth-based locally tangent frame is expressed as [8]:

(2) $${\dot{V}}^{n}=C_{b}^{n}f^{b}+g^{n}$$

Equation (2) gives gravity compensated acceleration in the n-frame. It is integrated numerically to get velocity and further integration will give the position in the n-frame (Equations (3) and (4)).

(3) $$V_{k}^{n}=V_{k-1}^{n}+\int_{k-1}^{k}{\dot{V}}^{n}(\tau )d\tau$$

(4) $$P_{k}^{n}=P_{k-1}^{n}+\int_{k-1}^{k}V^{n}(\tau )d\tau$$

The attitude update equation for foot-mounted PNS is driven by gyro inputs and is expressed in quaternion form in this work. Quaternion is a four-element vector representing rotation vector and magnitude of the rotation around that vector. The quaternion differential equation provides a relationship between the input angular rates and the quaternion rate, as shown in the flowing equation:

(5) $$\dot{q}=\frac{1}{2}q⊗{\tilde{\omega }}_{nb}^{b}\;∴{\tilde{\omega }}_{nb}^{b}\approx \omega_{ib}^{b}$$

where ${\tilde{\omega }}_{nb}^{b}$ is the quaternion matrix of the angular rates, which is function of gyro inputs, and the product symbol (⊗) represents the quaternion multiplication. If the rotation vector is written in skew-symmetric form, Equation (5) can be written in vector-matrix form as:

(6) $$\dot{q}=\frac{1}{2}[{\tilde{\omega }}_{nb}^{b}\times ]q\;∴[{\tilde{\omega }}_{nb}^{b}\times ]=\left[\begin{array}{cccc} 0 & -\omega_{x} & -\omega_{y} & -\omega_{z}\\ \omega_{x} & 0 & \omega_{z} & -\omega_{y}\\ \omega_{y} & -\omega_{z} & 0 & \omega_{x}\\ \omega_{z} & \omega_{y} & -\omega_{x} & 0 \end{array}\right]$$

where the components of rotation vectors come from gyro measurements. In discrete form, the Equation (6) is written as:

(7) $$\begin{array}{l} q_{k}=\left[\Delta \times \right]q_{k-1}\;∴\phi =\sqrt{\omega_{x}^{2}+\omega_{y}^{2}+\omega_{z}^{2}}\\ ∴\left[\Delta \right]={\left[\begin{array}{cccc} \cos(\phi /2) & \frac{1}{\phi }\sin(\phi /2)\phi_{x} & \frac{1}{\phi }\sin(\phi /2)\phi_{y} & \frac{1}{\phi }\sin(\phi /2)\phi_{z} \end{array}\right]}^{T} \end{array}$$

where [Δ×] is the skew-symmetric form of the vector [Δ]. The updated quaternion components are used to form the transformation matrix from body (b-frame) frame to n-frame, i.e.,

(8) $$C_{b}^{n}(k)=\left[\begin{array}{ccc} q_{0}^{2}+q_{1}^{2}-q_{2}^{2}-q_{3}^{2} & -2(q_{0}q_{3}-q_{1}q_{2}) & 2(q_{0}q_{2}+q_{1}q_{3})\\ 2(q_{0}q_{3}+q_{1}q_{2}) & q_{0}^{2}-q_{1}^{2}+q_{2}^{2}-q_{3}^{2} & -2(q_{0}q_{1}-q_{2}q_{3})\\ -2(q_{0}q_{2}-q_{1}q_{3}) & 2(q_{0}q_{1}+q_{2}q_{3}) & q_{0}^{2}-q_{1}^{2}-q_{2}^{2}+q_{3}^{2} \end{array}\right]$$

This transformation matrix ($C_{b}^{n}$) is used to transform the accelerometer sensed acceleration in b-frame to corresponding acceleration in n-frame, as given in Equation (2). Equations (3), (4) and (7) are used to propagate the PVA of the walker with only inputs from the foot-mounted IMU. In compact form, the INS mechanization equation is given in Equation (9) below:

(9) $$\begin{array}{l} \left[\begin{array}{c} P_{k}^{n}\\ v_{k}^{n}\\ C_{b,k}^{n} \end{array}\right]=\left[\begin{array}{c} P_{k-1}^{n}+v_{k-1}^{n}\Delta T\\ v_{k-1}^{n}+\left\{C_{b}^{n}f^{b}+g^{n}\right\}\Delta T\\ C_{b,k-1}^{n}+l(q,\omega_{ib}^{b},\Delta T) \end{array}\right]+\left[\begin{array}{c} w^{p}\\ w^{v}\\ w^{\psi } \end{array}\right]\\ ∴\;X_{k}=f(X_{k-1},f^{b},\omega_{ib}^{b},\Delta T)+w_{k} \end{array}$$

where l(.) is a function of gyro measurements, updated during the sampling time interval ΔT. It is mentioned in [6,16] that for best results in foot-mounted PNS applications, an IMU with high ranges of accelerometer (>10 g) and gyros (>900° per second) should be used, otherwise insufficient bandwidth will add systematic measurement/modeling errors in PNS.

### 2.2. Foot-Mounted INS Error Model

In order to estimate PVA errors, computed in INS stage, an error model must be used in Kalman filter estimator. Details of INS error model derivation are given in [11,15]. In a nutshell, the standard strapdown INS error-model is given below:

(10) $$\delta {\dot{v}}^{n}=\left[\delta \psi^{n}\times \right]f^{n}+C_{b}^{n}\delta f^{b}-2(\delta \omega_{in}^{n}\times v^{n}+\omega_{in}^{n}\times \delta v^{n})+\delta g^{n}$$

Since the navigation frame is fixed on the Earth, the error in the angular rate ($\delta \omega_{in}^{n}$) of the navigation frame is zero. For simplification, the gravity error ($\delta g^{n}$) is also assumed to be zero. For PNS application, the Coriolis acceleration caused by velocity error is also neglected here. Finally, after rearranging, INS error model for PNS application can be simplified as;

(11) $$\delta {\dot{v}}^{n}=C_{b}^{n}[f^{b}\times ]\delta \psi^{n}+C_{b}^{n}\delta f^{b}$$

where $\left[f^{b}\times \right]$ is skew-symmetric form of acceleration vector, $\delta \psi^{n}$ is the misalignment error vector and $\delta f^{b}$ is the accelerometer measurement error vector. Similarly, the position errors are modeled as:

Applying a similar assumption, the position error model for foot-mounted IMU is:

(12) $$\delta {\dot{P}}^{n}=\delta v^{n}$$

Finally, the attitude error model is expressed as a function of gyro measurement errors ($\delta \omega_{ib}^{b}$) only;

(13) $$\delta {\dot{\psi }}^{n}=-C_{b}^{n}\delta \omega_{ib}^{b}$$

Equations (11)–(13) form the INS error model to be used in Kalman filter in Section 5 to predict errors in PVA for foot-mounted IMU. In state space form, the INS error model is represented as:

(14) $$\begin{array}{l} \left[\begin{array}{c} \delta {\dot{P}}^{n}\\ \delta {\dot{v}}^{n}\\ \delta {\dot{\psi }}^{n} \end{array}\right]=\left[\begin{array}{ccc} 0_{3\times 3} & I_{3\times 3} & 0_{3\times 3}\\ 0_{3\times 3} & 0_{3\times 3} & -C_{b}^{n}[f^{b}\times ]\\ 0_{3\times 3} & 0_{3\times 3} & 0_{3\times 3} \end{array}\right]\left[\begin{array}{c} \delta P^{n}\\ \delta v^{n}\\ \delta \psi^{n} \end{array}\right]+\left[\begin{array}{cc} 0_{3\times 3} & 0_{3\times 3}\\ C_{b}^{n} & 0_{3\times 3}\\ 0_{3\times 3} & -C_{b}^{n} \end{array}\right]\left[\begin{array}{c} \delta f^{b}\\ \delta \omega_{ib}^{b} \end{array}\right]\\ \delta \dot{X}=A\delta X+BW\\ ∴\delta X^{n}\in {\mathbb{R}}^{9\times 1};\delta P^{n}\in {\mathbb{R}}^{3\times 1};\delta v^{n}\in {\mathbb{R}}^{3\times 1};\delta \psi^{n}\in {\mathbb{R}}^{3\times 1};W\in {\mathbb{R}}^{6\times 1};\delta f^{b}\in {\mathbb{R}}^{3\times 1};\delta \omega_{ib}^{b}\in {\mathbb{R}}^{3\times 1} \end{array}$$

The process noise, which consists of IMU zero-mean white noise sequences, has the covariance matrix Q defined by:

(15) $$Q=Ε\{W_{n}W_{n}^{T}\}=\left[\begin{array}{cc} \sigma_{f^{b}}^{2} & 0_{3\times 3}\\ 0_{3\times 3} & \sigma_{\omega^{b}}^{2} \end{array}\right]\in {\mathbb{R}}^{6\times 6}$$

where $\sigma_{f^{b}}^{2},\sigma_{\omega^{b}}^{2}$ are variances of IMU. The discrete-time counterparts of matrices (A,B,Q) are obtained as follows:

(16) $$\begin{array}{l} F=I+A\Delta T+\frac{1}{2}(A\Delta T)\\ G=B\Delta T;\;Q_{d}=BQB^{T} \end{array}$$

If accelerometer and gyro bias vectors are also included in the state vector, then these terms are also augmented in the state space model. However, as mentioned in [8,9], these are no major error sources distorting the IMU output during high dynamic motions that the foot-mounted IMU is exposed to. Hence, IMU bias error states are not included in the EKF framework in this study. However, initial gyro bias should be estimated by averaging stationary data for a few seconds (>10 s) before staring the experiment. This initial gyro bias must be subtracted from gyroscope readings for each axis before utilizing them in the PNS algorithm.

## 3. INS Aided with Motion Constraints (“*Virtual Sensors”*)

The aim of this study is to use only a single IMU as a motion sensor for pedestrian navigation in an unstructured environment and exploit the motion constraints of the walker to reduce drift in the INS solution. Therefore, we will develop algorithmic “*virtual sensors*” which will constrain the INS errors as the agent walks or stops for long time during walking.

### 3.1. Zero-Velocity Update (ZUPT)

It is a natural process for human and legged animals as well that when they walk in a normal way, each time they push the ground backwards with their feet, and at least one of their feet remains stationary for a moment during ground contact. In other words, the velocity of that foot momentarily becomes close to zero. If an IMU is mounted on that foot, it also experiences brief cyclic stance phases during walking. If this zero-velocity moment is detected (algorithmically or by using contact sensors), this information may be fed to the Kalman filter estimator to estimate the accumulated errors in PVA since the last update of such knowledge. Since a walking pattern repeats itself rapidly depending on the walking speed, the zero-velocity updates are applied as “*pseudo-measurements”* to the Extended Kalman filter [2], which utilizes these measurements as correct knowledge and estimates the errors in PVA as its output.

### 3.2. Zero Velocity Detection Algorithm

To detect the stance phases during walk, one can use actual contact sensors which sense the pressure exerted by the foot on the ground when it pushes the ground backwards during stance phase [17,18]. However, as the main idea of this paper is to use only a single 9DoF IMU, IMU signals are used to detect the stationary periods during walking. There are four commonly used methods or their combinations [3,19] in the literature for zero velocity detection. These are: Acceleration Moving Variance Detector, Acceleration Magnitude Detector, Stance Hypothesis Optimal Detector (SHOE) and Angular Rate Energy Detector (ARE). However, [20] ARE and SHOE have been shown to perform the best in experiments involving different gait patterns [19,21,22]. Here, we use ARE for stance detection in this paper. A brief description of ARE is given as follows:

(17) $$\begin{array}{l} T(z_{n}^{\omega_{ib}^{b}})=\frac{1}{W\sigma_{\omega_{ib}^{b}}^{2}}{\sum_{k=n}^{n+W-1}\left\|y_{k}^{\omega_{ib}^{b}}\right\|}^{2};\\ ∴{\left\|y_{k}^{\omega_{ib}^{b}}\right\|}^{2}=\sqrt{{\left(\omega_{ib,x}^{b}\right)}^{2}+{\left(\omega_{ib,y}^{b}\right)}^{2}+{\left(\omega_{ib,z}^{b}\right)}^{2}} \end{array}$$

where *W* is window size for weighted average of gyro signals ($y_{k}^{\omega_{ib}^{b}}$). T(.) is the test statics of the detector. Walking patterns are detected as:

(18) $$Walk\;pattern(P_{i})=\left\{ \begin{array}{l} Stance\;phase:if(T(z_{n}^{\omega_{ib}^{b}})<\gamma )\\ Stridephase:\;\mathrm{otherwise} \end{array}\right.$$

**Figure 2:**
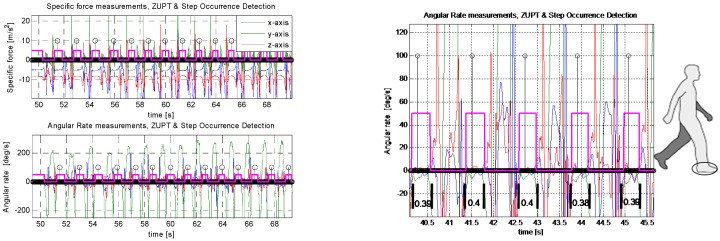
(**Left**) ZUPT detection and step occurrence during normal walking. The zero-velocity periods, as foot falls on the ground, are shown as a pink square wave and foot count is shown by a small circle (black), overlaid on the accelerometer and gyro signals; (**Right**) Zoom-in of some steps to show the stance period (~0.4 s) in a typical walk.

The zero-velocity detection periods and foot stance count are shown in Figure 2, overlaid on the accelerometer and gyro signals during normal walking. A few steps are displayed in the lower part of this figure to show the stance time period in seconds. The average stance period during normal walking is about 0.4 s, as reported in [15,16].

The threshold (γ) is optimized based on experiments. However, this issue is not discussed in this paper.

### 3.3. Why Is a Zero Velocity Update Important?

The error growth in position is cubic with time while using only IMU, but this rapid accumulation of error can be mitigated by frequently aiding the IMU with ZUPT in the EKF framework [10] in PNS application. The ZUPT breaks this cubic error growth by aiding after about every 0.1~0.3 s and compensates for most of the errors of PVA and IMU [5,6,12]. However, the heading error and the heading gyro bias are the only important states which are not observable by applying ZUPTs [16]. The relationship between acceleration errors and attitude errors in the n-frame is shown in Equation (11), which can further be represented in component form for clarity [23,24]:

(19) $$\begin{array}{l} \delta {\dot{v}}^{n}=C_{b}^{n}[f^{b}\times ]\delta \psi^{n}+C_{b}^{n}\delta f^{b}\\ \delta {\dot{v}}^{n}=[f^{n}\times ]\delta \psi^{n}+\delta f^{n}\\ \left[\begin{array}{c} \delta {\dot{v}}_{x}^{n}\\ \delta {\dot{v}}_{y}^{n}\\ \delta {\dot{v}}_{z}^{n} \end{array}\right]=\left[\begin{array}{ccc} 0 & -f_{D} & f_{E}\\ f_{D} & 0 & -f_{N}\\ -f_{E} & f_{N} & 0 \end{array}\right]\left[\begin{array}{c} \delta \phi \\ \delta \theta \\ \delta \psi \end{array}\right]+\left[\begin{array}{c} \delta f_{x}^{n}\\ \delta f_{y}^{n}\\ \delta f_{z}^{n} \end{array}\right]\\ \delta {\dot{v}}_{x}^{n}=-f_{D}\delta \theta +f_{E}\delta \psi +\delta f_{x}^{n}≅-f_{D}\delta \theta +\delta f_{x}^{n}\\ \delta {\dot{v}}_{y}^{n}=f_{D}\delta \phi -f_{N}\delta \psi +\delta f_{y}^{n}≅f_{D}\delta \phi +\delta f_{y}^{n}\\ \delta {\dot{v}}_{z}^{n}=-f_{E}\delta \phi +f_{N}\delta \theta +\delta f_{z}^{n}≅\delta f_{z}^{n} \end{array}$$

Here, $f_{N,E,D}$ is the acceleration in North, East, Down axis of the n-frame. Equation (19) shows that velocity error is a function of acceleration measurement and error in attitude angles. In ZUPT-aided INS, we use the velocity error as measurement in the stationary phase of the foot to correct PVA errors in EKF. During ZUPT, the horizontal forces in the local level frame are essentially zero $(i.e.,f_{N}=f_{E}\approx 0)\;$ and specific force $f_{D}$ in downward direction is approximately equal to gravity constant. Therefore, from Equation (19), it is seen that the velocity errors in North (x-axis) and East (y-axis) directions are only related to errors in roll and pitch angles through a specific force $f_{D}$ in the downwards direction. Conceptually, this means that during ZUPT period, the dynamic change in horizontal velocity is proportional to the change in roll and pitch errors. Improving the velocity estimation through ZUPT means that roll and pitch errors are improved as well but heading errors are not improved at all by ZUPT [12,16,24]. Hence, we have to use other methods to reduce drift in heading errors, and hence the trajectory cross-track errors are reduced. For drift reduction in heading, we have used some additional motion constraints of the pedestrian gait patterns such as moving along straight paths, standing still for a long time, etc.

### 3.4. Walking along Straight Paths: Straight Trajectory Heading (STH) Update

People walk along straight paths, most of the time, in order to save time and energy. Also, most corridors and hall ways are constructed straight in conventional buildings. When the user walks along these straight paths, this situation can be detected and exploited to reduce errors in gyro heading angle [3,25]. In [25], the authors proposed a complicated process, utilizing feedback control theory, to reduce z-gyro drift. A simple mechanism is used in this work, in the n-frame domain, to distinguish the near straight path from the curved path using the change in average heading angle during consecutive last n-footsteps in stance phase, i.e.,

(20) $$\Delta \psi_{k}=\psi_{k}-\frac{1}{n}\sum_{s=1}^{n}\psi_{k-s}$$

If $\Delta \psi_{k}>th.$, then we assume that the user is walking along a straight path and hence apply straight trajectory heading (STH) update. Empirically, we have chosen number of steps in STH algorithm as *n* = 3, and $\Delta \psi_{k}<5$°.

**Figure 3:**
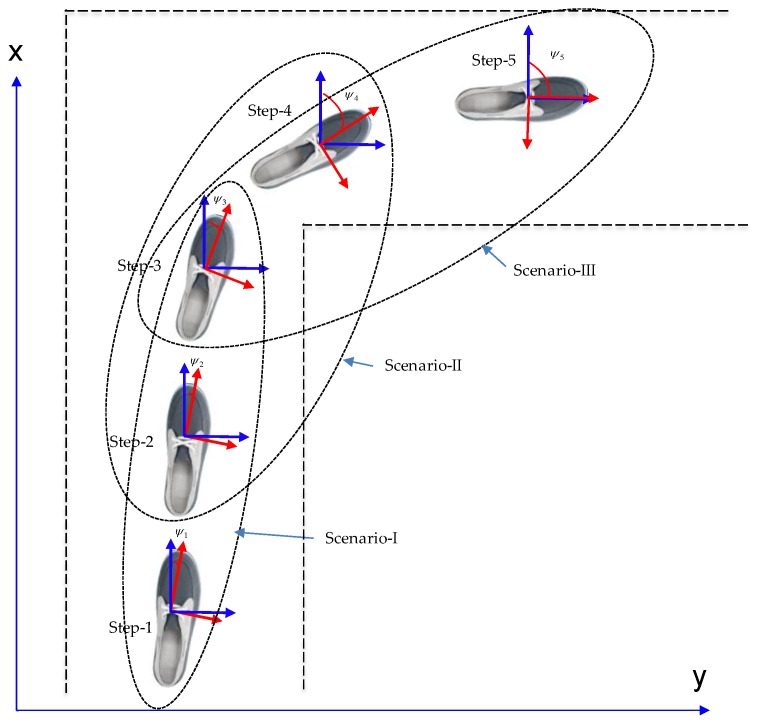
Illustration of walking along a straight path in a typical indoor corridor.

**Figure 4:**
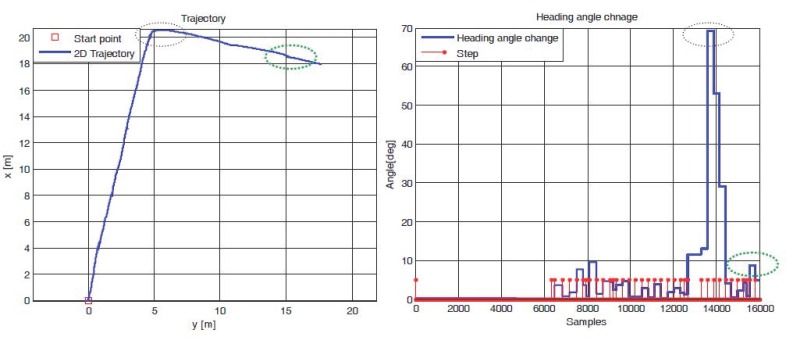
Experimental data for walking along a typical corridor. (**Left**) 2D trajectory in corridor; (**Right**) Corresponding change in heading angle at each foot step.

In a normal walk, three steps seem to be minimum number of steps to execute direction change and check the heading angle change in each detected step, as illustrated in Figure 3 below. We can include one previous and one further step too, but it will be redundant and merely increase the computational cost in the algorithm. For instance, in scenario-I, the pedestrian is walking along a typical indoor corridor with a nearly straight walk. The heading angle change in each consecutive step is less than about five degrees in the straight corridor walk, as confirmed by experimental data in Figure 4. However, when the user takes a right/left turn in a corridor, as most typical building have corridors at 90°, three steps in a window show an obvious change in heading angle (as illustrated in scenario-II and scenario-III in Figure 3), which in shown by experimental data in Figure 4.

Figure 4 shows experimental data for walking along a typical building corridor with one right turn. The black dotted circle shows a right turn during walking and the green dotted circle represents little change in walking direction, even in the same corridor. In both cases, the STH algorithm detects the change in walking direction (with a threshold of five degrees) and rejects the heading angle update in the EKF algorithm if the heading angle change exceeds the threshold. This way, drift from the estimated trajectory is reduced by applying STH continuously in the PNS algorithm. Further results are shown in Section 6.3 for *Indoor Environment tests.*

### 3.5. Standing Still for a Long Time: PA Locking Mechanism

**Figure 5:**
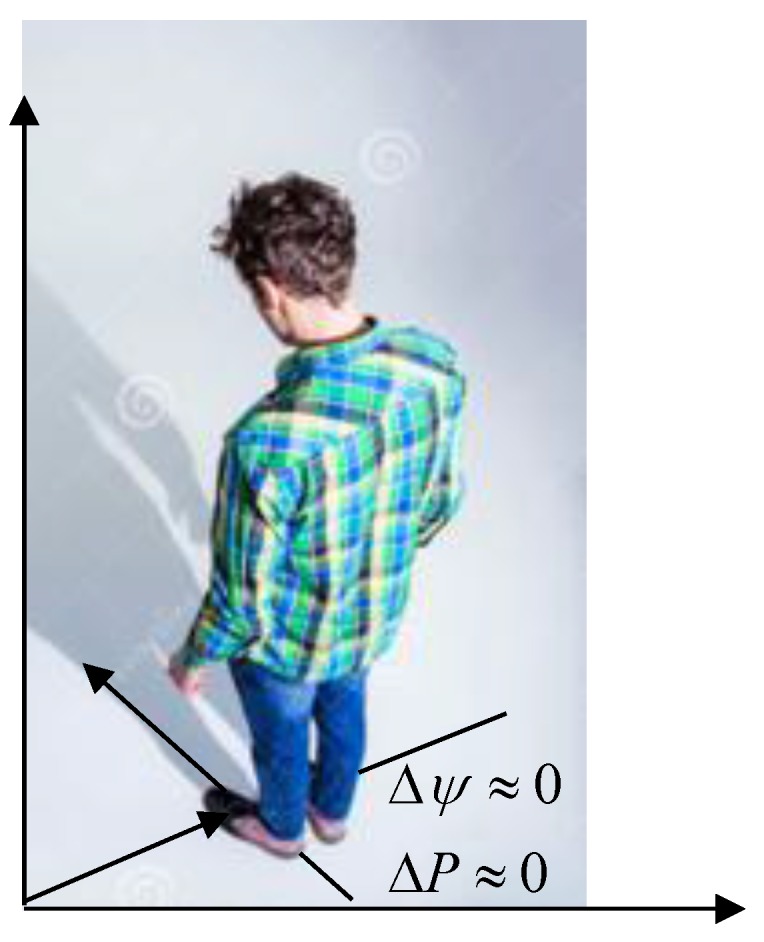
Standing-still condition, whereby both position and heading should not be changing.

People are not always walking consistently, and they have to stop at times during normal walking. It has been observed that ZUPT does not guarantee complete stand-still conditions [26]. Hence, we must devise another method to guarantee non-drift of PVA during complete stand still. We adopted a rather simple method that is tested experimentally and effectively reduces error in position and attitude. When zero-velocity is detected, we check the forward velocity during this period; if it remains at zero for an extended period of time e.g., more than 5 s, it means that the walker has stopped moving forward and is in a complete stand-still condition. If this condition is met, we apply a simple rule to ‘*lock*’ the position and attitude (quaternion) at their current state, i.e.,

(21) $$\begin{array}{l} if\;v_{x}=0\;for\;Time>thrs,\;∴thrs=5\sec.\\ \;P_{k}=P_{k-1}\;∴P_{k}\in {\mathbb{R}}^{3\times 1}\\ \;q_{k}=\frac{q_{k-1}}{10^{6}}\;∴q_{k}\in {\mathbb{R}}^{4\times 1}\\ end \end{array}$$

i.e., position remains the same and change in quaternion is negligible, as illustrated in Figure 5.

Stance phase periods during normal walking vary depending on walking style/speed, age, terrain and other factors. We have observed that stance period is about ~0.4 s, as observed in Figure 2 above. Many other authors report different values [5,6,8]. However, to ensure a complete stand-still condition, some longer and safer stance time periods (higher than about 1 s) are needed. We have chosen 5 s to insure that the user is no longer walking and has completely stopped. This value can be 3 s, or 4 s. It will not affect the accuracy of the STH mechanism, so 5 s is a reasonable value to choose.

Until now, only motion constraints are discussed to reduce error of INS. In the next sections, we will present the technique to use 3D magnetometer’s “*healthy*” data, suitable to aid INS with absolute heading angle.

## 4. Absolute Heading Estimation Using Magnetometer

### 4.1. Magnetometer Used as Digital Compass

The Earth’s magnetic field vector has three components ($H_{x},H_{y},H_{z}$), which can be measured by 3D magnetometers. The $H_{x}$ component is in the direction of motion of the sensor and $H_{y}$ is perpendicular to it in the right-hand direction. Using these two horizontal components, a combined horizontal component ($H_{xy}=\sqrt{H_{x}^{2}+H_{y}^{2}}$) of the Earth’s magnetic field is obtained. This horizontal component is always parallel to the Earth’s surface and aligns itself to the magnetic North–South direction. The magnetic heading angle is obtained from horizontal components, as follows:

(22) $$\psi_{mag}=\tan^{-1}(\frac{H_{y}}{H_{x}})$$

**Figure 6:**
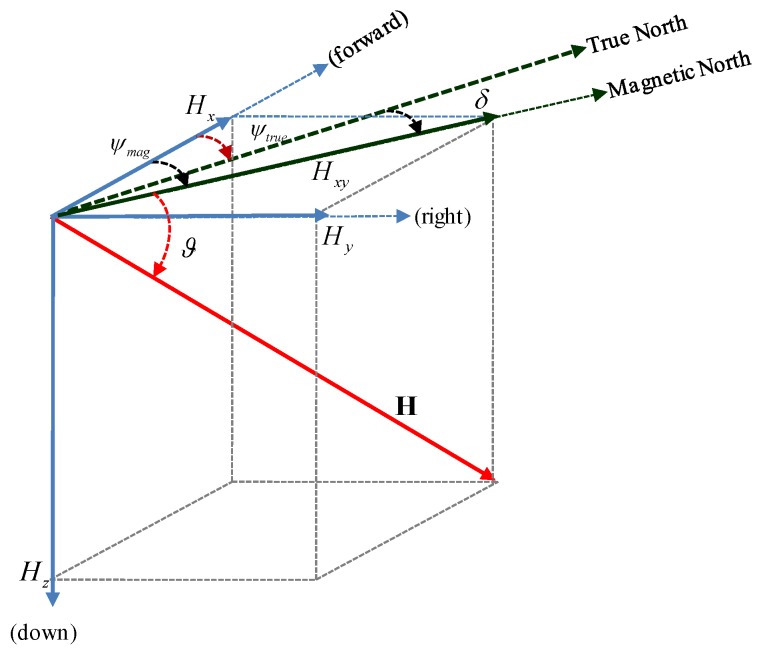
Earth’s magnetic field and calculation. ϑ is the dip-angle, δ is declination angle, $\psi_{mag}$ magnetic heading and $\psi_{true}$ is true heading with respect to true North.

The 3D Earth’s magnetic field vector and its relationship with the magnetic heading angle is shown in Figure 6.

The true heading angle and magnetic heading have some offsets, depending on the location on the Earth’s surface. This offset is called *Magnetic declination* (δ), which is taken as positive in an eastward direction and negative in a westward direction. Then, the true heading of the vehicle is obtained by subtracting the declination angle from magnetic heading, i.e.,

(23) $$\psi_{true}=\psi_{mag}-\delta$$

**Figure 7:**
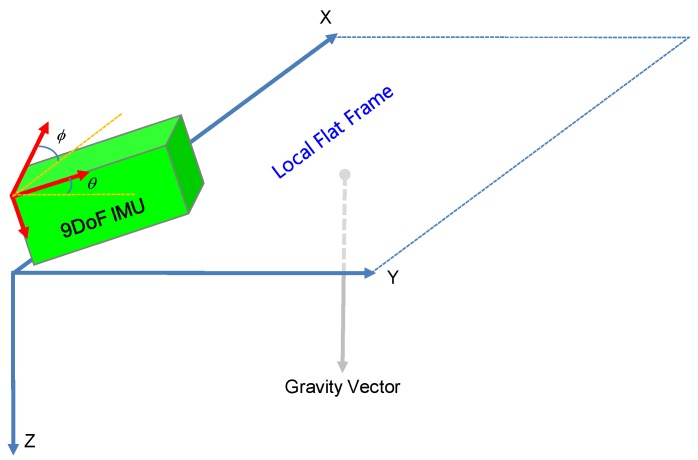
Compass frame (red) orientation with respect to the Earth’s surface. Local flat frame (blue) is paralell to the Earth’s surface and ϕ ,θ are roll and pitch angles, respectively.

It is crucial to obtain the horizontal magnetic field components as accurately as possible, otherwise the calculated heading angle will be contaminated with tilt errors. In most applications, it is impractical to assume roll and pitch angles of the magnetometer sensor as zero. Hence, we have to compensate the magnetometer readings with the title angles (roll, pitch) to obtain horizontal components from it (Figure 7).

The measurements of the 3D magnetometer should be transferred to the Earth’s fixed plane frame, so that the horizontal components of magnetic field are obtained to calculate magnetic heading. The roll angle and pitch angle are obtained, in our application, from the output of the designed attitude module (as tilt angles are not affected by magnetometer data). Then, the title compensated horizontal components of Earth’s magnetic field are obtained as;

(24) $$\begin{array}{l} H_{x}=m_{x}\cos(\theta )+m_{y}\sin(\phi )\sin(\theta )+m_{z}\cos(\phi )\sin(\theta )\\ H_{y}=m_{y}\cos(\phi )-m_{z}\sin(\phi ) \end{array}$$

where ($m_{x},m_{y},m_{z}$) is magnetometer output in the sensor frame. Earth’s magnetic field is measured in units of *Tesla* *(T)* or *Gauss (G).* Note that 1 T = 10,000 G.

### 4.2. Transient Magnetic Anomaly Detection (MAD) and Compensation Algorithm

**Figure 8:**
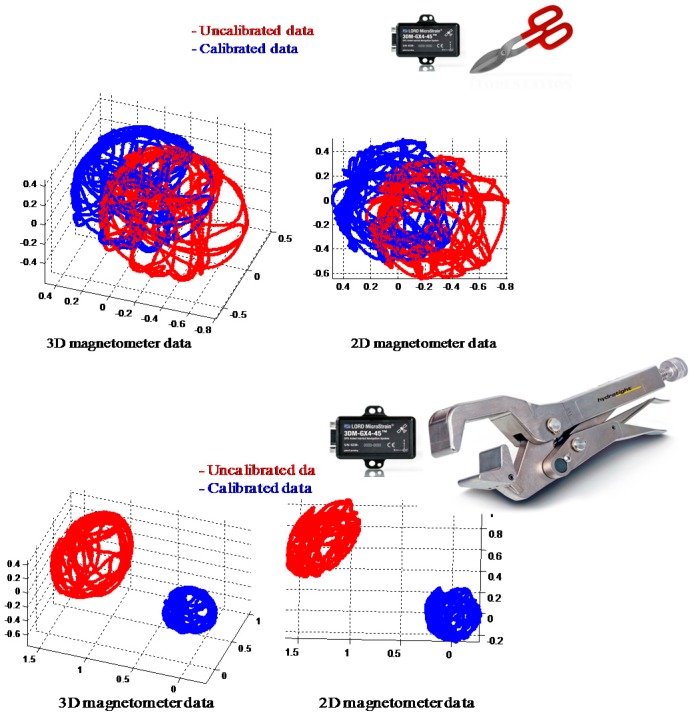
Local magnetic interference calibration: Magnetometer data before and after calibration in mild magnetic distortion environment (**Top**) and strong local distortion (**Lower**) present in the calibration environment. The 3D sphere and 2D circle are centered at (0, 0) after hard and soft iron calibration.

Magnetic interference can be categorized into two types: local magnetic interference and transient magnetic distortion. Local magnetic interference is caused by the installation of a magnetometer sensor on a platform having ferromagnetic material and current carrying wires, etc. This magnetic interference/distortion falls in one of the two categories; *hard or soft iron* *distortion*. Hard iron distortions are created by ferromagnetic materials with permanent magnetic fields e.g., magnets, speakers. They are time invariant. If the piece of magnetic material is physically attached to the same reference frame as the sensor, then this type of hard iron distortion will cause a permanent bias in the sensor output. Soft iron distortions are considered deflections or alterations in the existing magnetic field. It is caused by materials which can become magnetized and produce time varying magnetic fields, e.g., current carrying traces on the PCB, steel shields, batteries or other magnetically soft materials [27]. This type of distortion is commonly caused by metals such as nickel and iron. In most cases, hard iron distortions will have a much larger contribution to the total uncorrected error than soft iron. However, local magnetic interference can easily be compensated by employing well-known calibration procedures [27,28]. Results of local magnetic interference calibration are shown in Figure 8 below.

The other type of magnetic interference is called *transient distortions* which are produced when the sensor comes into the vicinity of some ferromagnetic material during operation. For instance, in the indoor environment, considered as a harsh environment in PNS application, is usually full of ferromagnetic materials, e.g., iron, steel, power lines, electrical machines, etc. Hence, the magnetometer output is badly affected when it comes into the vicinity of such ferromagnetic materials or permanent magnets [28]. Therefore, it is common practice not to use magnetometers for heading calculation in such transient magnetic interference environments. However, the validity of data which the magnetometer provides is far higher for navigation purposes as absolute heading can be very easily calculated using this sensor, if magnetometer data is free from distortions. Hence, there are efforts being made to use magnetometer data in such unfriendly environments too by designing such algorithms which can provide some detection and compensation in heading estimation. One such algorithm is proposed in this study, called *Magnetic Anomaly Detection (MAD)* and compensation algorithm. The main crux of the idea is that the *magnetic field strength* and its direction from the ground, called *dip angle*, remain almost constant in a local area within a few kilometers in a magnetically clean environment. If we take these factors into consideration and compare our measured magnetic field strength and calculated dip angle with these reference values, we can easily detect any anomaly in the magnetometer readings. If we take the mean of dip-angle and magnitude of magnetic field at starting time, assuming no motion and distortion during the starting stationary period, then any further anomalies can be detected easily. The magnetic field strength is simply the magnitude of the three components of magnetometers and dip angle is calculated using magnetometer data alone, as shown in Equations (25) and (26).

(25) $$H=\sqrt{h_{x}^{2}+h_{y}^{2}+h_{z}^{2}}$$

The dip-angle calculated using magnetometer data (in n-frame) is given as [29]:

(26) $$\alpha =\tan^{-1}(\frac{h_{z}}{\sqrt{h_{x}^{2}+h_{y}^{2}}})$$

## 5. EKF Algorithm for Foot-Mounted PNS

The final step is to fuse all valid motion information of the walker in an estimator in order to reduce error drifts in the INS only solution. We have developed necessary models for the EKF framework in previous sections, and now formally present the prediction and update part of the EKF estimator for PNS application in this paper. In a discrete time domain, the EKF prediction and update cycles are summarized here:

(i) Initialize the filter as follows:
  (27) $$\begin{array}{l} \delta {\hat{x}}_{0}^{+}=E(\delta x_{0})\\ P_{0}^{+}=E[(\delta x_{0}-\delta {\hat{x}}_{0}^{+}){\left(\delta x_{0}-\delta {\hat{x}}_{0}^{+}\right)}^{T}] \end{array}$$
  For *k =* 1, 2, …., *N*, perform the following:
(ii) Obtain discrete state transition matrices:
  (28) $$\begin{array}{l} F=I+A\Delta T+\frac{1}{2}{\left(A\Delta T\right)}^{2}\\ G=B\Delta T;\;Q=BQ_{c}B^{T} \end{array}$$
(iii) Perform the propagation step:
  (29) $$\begin{array}{l} \delta {\hat{x}}_{k}^{-}=0\\ P_{k}^{-}=F_{k-1}P_{k-1}^{+}F_{k-1}^{T}+G_{k-1}Q_{k-1}G_{k-1}^{T} \end{array}$$
(iv) Perform the measurement update step if:
  - ZUPT, PA-Lock, STH condition are true.
  - *MAD* condition is true.
  (30-i) $$k_{k}=P_{k}^{-}H_{k}^{T}{\left(H_{k}P_{k}^{-}H_{k}^{T}+R_{k}\right)}^{-1}$$
  (30-ii) $$\delta {\hat{x}}_{k}^{+}=\delta {\hat{x}}_{k}^{-}+k_{k}[z_{k}^{n}-h(\delta {\hat{x}}_{k}^{-},0)]\equiv k_{k}[\delta z_{k}^{n}]$$
  (30-iii) $$P_{k}^{+}=(I-k_{k}H_{k})P_{k}^{-}$$
(v) Feedback error correction of PVA:
  (31) $$\begin{array}{l} {}^{c}P_{k}^{n}=P_{k}^{n}-\delta {\hat{x}}_{k,P}^{+}\\ {}^{c}v_{k}^{n}=v_{k}^{n}-\delta {\hat{x}}_{k,V}^{+}\\ {}^{c}C_{b,k}^{n}={\left(I-[\delta {\hat{x}}_{k,\psi }^{+}\times ]\right)}^{-1}C_{b,k}^{n} \end{array}$$

**Figure 9:**
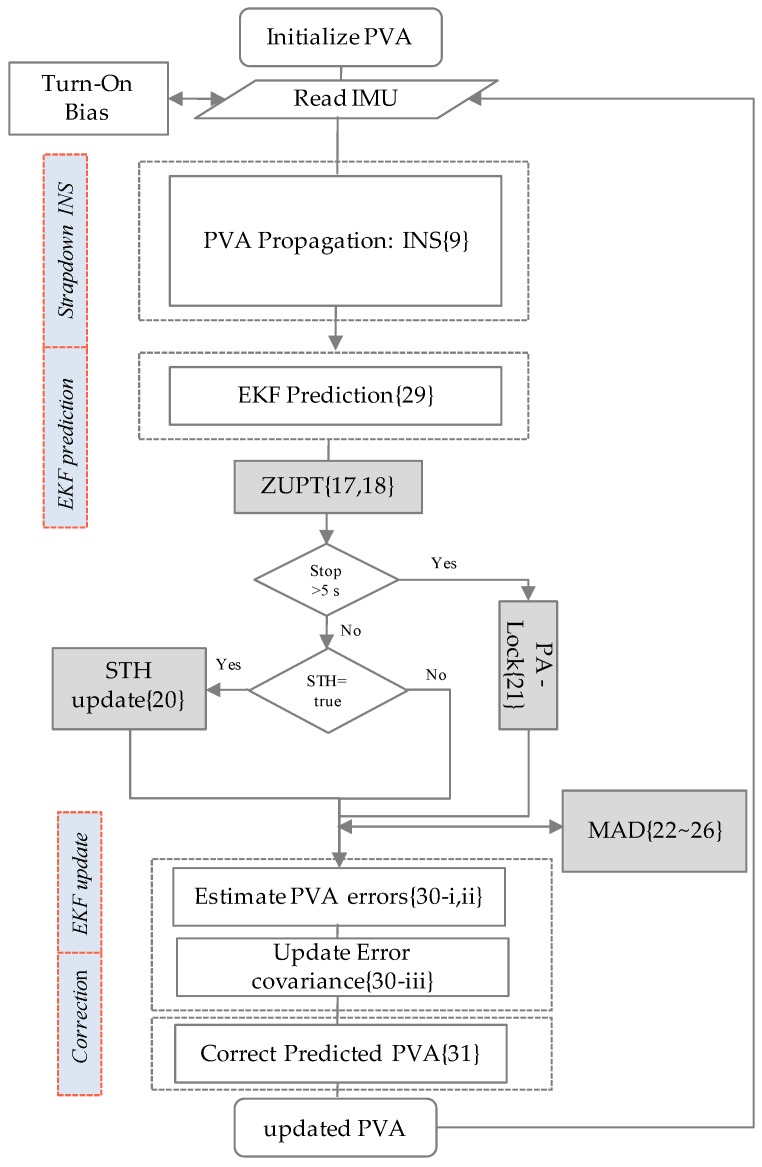
EKF algorithm flowchart of PNS: Data fusion of INS, ZUPT, STH, PA-lock and *MAD*.

The flowchart of proposed fusion algorithm if INS is aided with motion constraints (i.e., ZUPTs, PA-Lock, STH) and 3D magnetometer data is shown in Figure 9 below.

## 6. Experimental Results and Discussion

In this section, we provide experimental results for PNS design and applications in harsh environments, like the indoors, urban canyons, and forest environments. First, we define our experimental set-up and result verification method, and then present the results obtained in graphical and numerical form.

### 6.1. Test Environments

**Figure 10:**
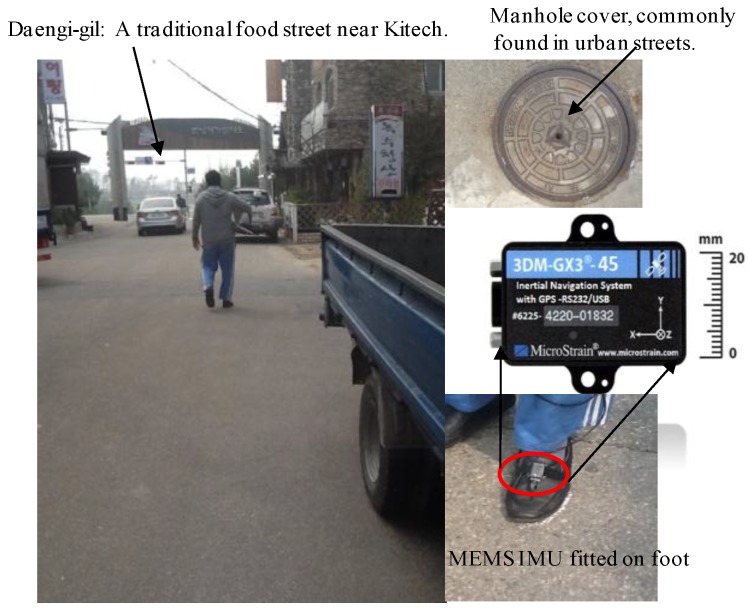
Urban canyon test environment; Daengi-gil, a food-street near Kitech. The IMU sensor is fitted on top of the left foot. In the upper right corner, a large metallic manhole cover, found in many urban streets, along with other large metallic objects are shown.

**Figure 11:**
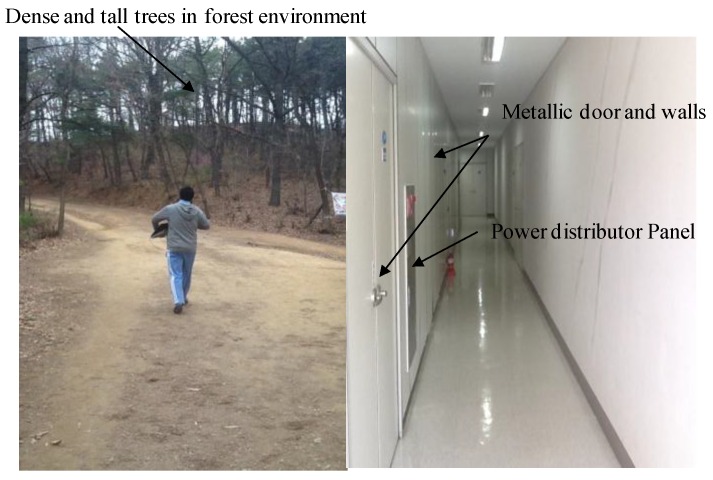
Forest environment (**Left**) and typical indoor environment (**Right**). Both environments fall into the GPS-denied and magnetically distorted environment category.

The environments chosen to validate the proposed algorithms are such that they fit in the category of “*Navigationally Harsh Environment”* [30]. For example, we choose a nearby street as an urban canyon, Figure 10. In such kinds of environments, GPS signals may not be completely blocked, but they may have large reflections, thereby reducing the availability of GPS for reliable navigation. Similarly, a walking test was conducted in a forest, which is covered by tall and dense trees. The GPS signals are vulnerable in such environments. The third candidate of harsh environments is the indoors, Figure 11, with no pre-installed navigation infrastructures, i.e., R.F, Ultrasonic signals, visual land marks, etc. Both absolute navigation systems/sensors, e.g., GPS and magnetometer, are prone to large errors in such environments, and hence normally cannot be used.

### 6.2. Experimental Setup and Evaluation Method

Experiments were conducted in both indoor and outdoor environments, with no prior information about the test environment and the user itself. The IMU sensor (3DM-GX3-45) from Microstrain Inc. [31] was used for data collection in all experiments. Although this IMU provides many kinds of navigation data, we picked up only raw accelerometer, gyro and magnetometer 3D data and the rest of the navigation solution and data processing was done using our own designed fusion algorithm. The IMU was fitted on the left foot and the user walked at a normal walking speed. In some experiments, the user also stood still for more than 5 s to check the effect of the PA-locking technique. For estimation evaluation, we have chosen root-mean-square error (RMSE) as an accuracy measure in this work. It is used to measure the difference between actual values and the output of an estimator. For quantitative comparison, we checked only the starting and final positions, both in 2D and 3D, in all walking tests. We assume starting position as (0, 0, 0), hence, only the final estimates are inserted into the RMSE formula. The other quantitative quality indicator is the *error per* 100 m (i.e., percentage error of the total distance travelled). Both of these quality indicators are given in comparison tables.

### 6.3. Static Test to Check PA-Lock Effectiveness

**Figure 12:**
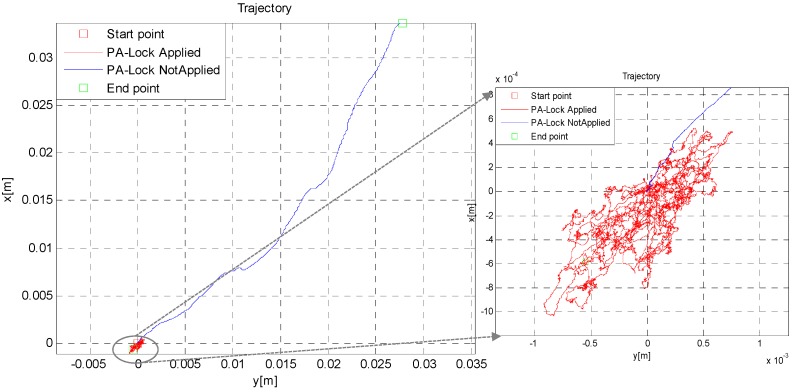
2D Position error reduction by using PA-locking technique.

**Figure 13:**
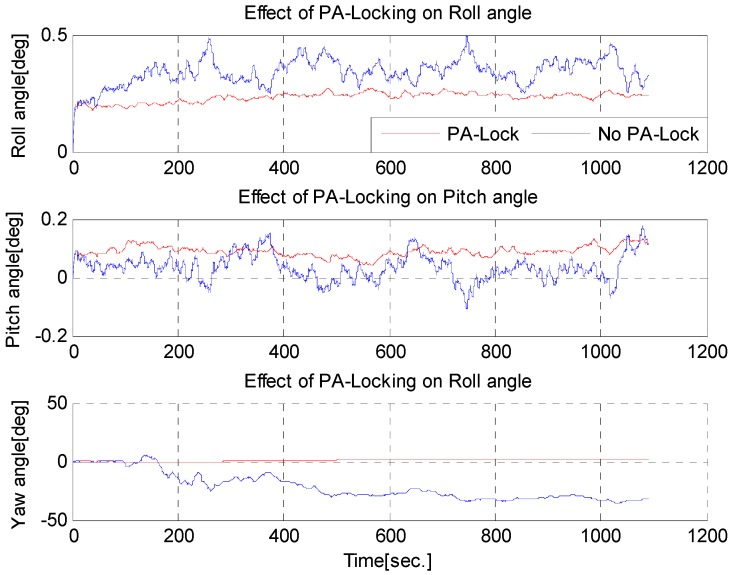
Roll, pitch and heading error reduction with PA-locking technique.

The practical benefits of simple PA-locking mechanism to reduce drift in position and attitude is demonstrated in Figure 12 and Figure 13. The numerical results are given in Table 1 and Table 2. Note that drift reduction in heading angle is approximately 15-fold. This huge drift reduction is due to the fact that, in simple INS or ZUPT aided INS (*Z-INS*), the heading angle is not observable and its error increases unbounded. ZUPT only constrains roll and pitch error growth, but not the heading error [23]. However, by constraining the change in attitude quaternion when the standing-still condition is met, this drift in heading angle can be drastically reduced.

### 6.4. Indoor Environment Tests

**Figure 14:**
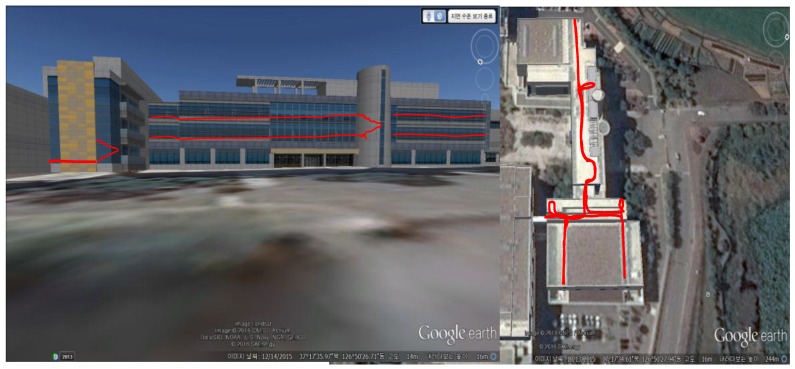
Multi-floor indoor test environment. For ease of visualization, an approximate reference path manually drawn on the building image (front and top view) was taken from Google Earth.

**Figure 15:**
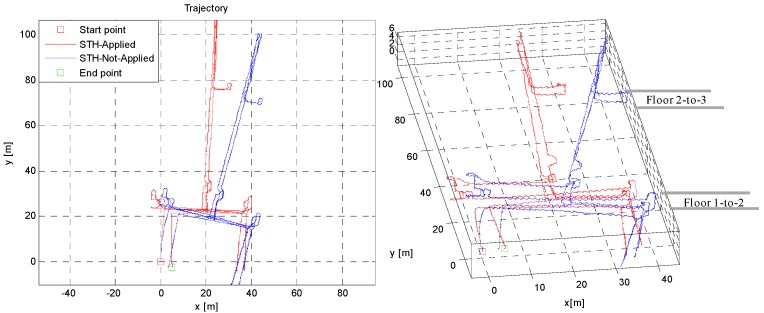
2D and 3D trajectory plot comparison in indoor test. Effect of STH applied is clearly seen in red trajectory, which has less cross-track divergence.

The indoor test was conducted in a three-floor building in KITECH, with double flight stairs. As most of the building has straight corridors and hallways, aiding Z-INS with STH should be more effective in such a building environment. In Figure 14, the approximate reference path is drawn manually in 2D and 3D for visual inspection; In Figure 15, the red trajectory (STH aided estimates) keeps the parallelism and perpendicularity with the building layout, but the blue trajectory (only ZUPT aided INS) is not aligned with the building layout. The STH un-aided trajectory has drift, especially in heading. The simple technique, i.e., STH, greatly reduces the heading drift in this indoor environment test. It shows the effectiveness of the STH method applied, without using any other sensor to reduce errors in the overall localization in unstructured environments.

The numerical results are given in Table 3 below. Simple STH application not only reduces 2D errors in trajectory, but reduces drift in height as well. The overall position error is less than 1% of the total distance traveled.

### 6.5. Outdoor Tests: Urban Canyon Environment Test

**Figure 16:**
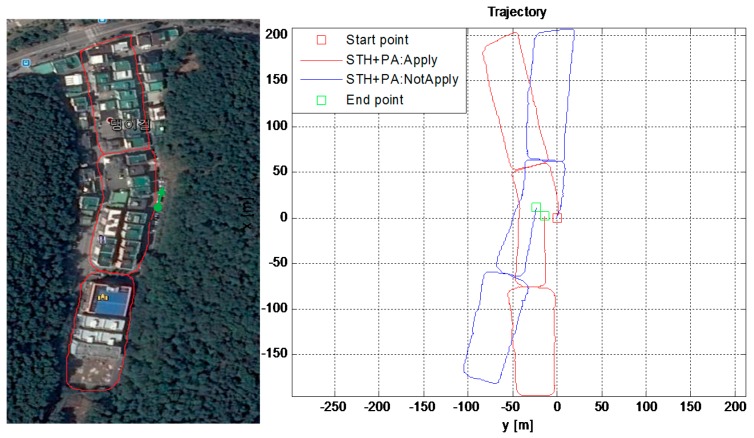
Urban environment test: **Left**: The reference path is manually drawn on the image taken from Google Earth. **Right**: The estimated trajectories obtained from the proposed algorithm.

To test the proposed techniques, using virtual sensors, for reducing drift in ZUPT aided INS, we conducted one long-time test in the urban environment, as shown in Figure 16. In such an environment, GPS signals are vulnerable to blockage and/or large reflections due to tall buildings and narrow passages. The start and end point was the same while performing this test and the final position errors are calculated to verify the improvement made by the proposed technique. During this long test, at some points, the user rested for more than a minute and, hence, the PA-locking mechanism is applied as well. It is shown in Section 3.5 that in the standing-still condition, ZUPT alone cannot constrain the drift in position and attitude. Hence, when the user stood still for more than 5 s, the PA-locking technique was applied in this work.

The numerical results are compared in Table 4 below. Simple STH and PA-locking techniques reduce the drift in ZUPT aided PNS. The overall position error is less than 2% of the total distance traveled in this long (1 km) urban test which took about 45 min. The position estimates using single IMU aided with motion constraints only are impressive.

### 6.6. Forest Canopy Environment Test

**Figure 17:**
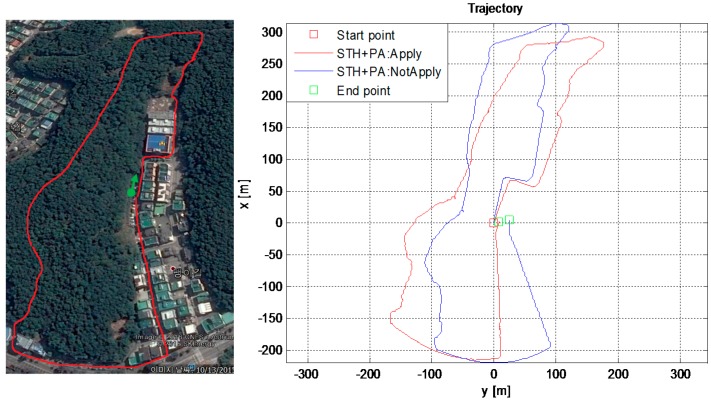
Forest environment test: (**Left**) The reference path is manually drawn on the image taken from Google Earth; (**Right)** The estimated trajectory output by the proposed algorithm.

To validate the proposed scheme, i.e., reducing drift in ZUPT aided INS using virtual sensors, we conducted one long-time test in a dense forest environment, categorized as one of the harsh environments in this work. In such an environment, GPS signals are vulnerable to blockage and/or reflections due to forest canopy; hence, GPS cannot be used for reliable positioning in such harsh environments. For visual verification, the reference trajectory drawn manually, taken during the test in the forest, is shown in the left half of Figure 17. The estimated trajectory with (red) and without (blue) using STH and PA-locking technique is shown in the right half of Figure 17. It is observed that STH and PA-Lock have a positive impact on position estimation accuracy as compared to only ZUPTs to EKF, as the trajectory shape is more similar to the actual path taken during this experiment. A quantitative comparison in terms of RMS error is given in Table 5 below.

Again, the numerical results reveal that by simply applying these two extra motion constraints (i.e., *STH and PA-Locking*) reduces the drift in ZUPT aided PNS. The overall position error is about 1% of the total distance traveled in this long (>1 km) forest test which took about 40 min. This test too confirms the viability of our proposed algorithm to reduce drift in INS in this PNS application.

## 7. Experimental Set-up and Evaluation Method for *MAD* Algorithm

### 7.1. Static Test

**Figure 18:**
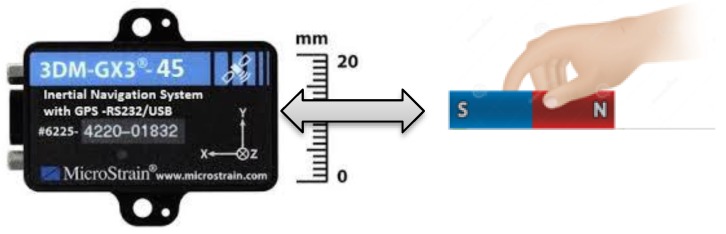
Experimental set-up for *MAD* algorithm verification in static test.

**Figure 19:**
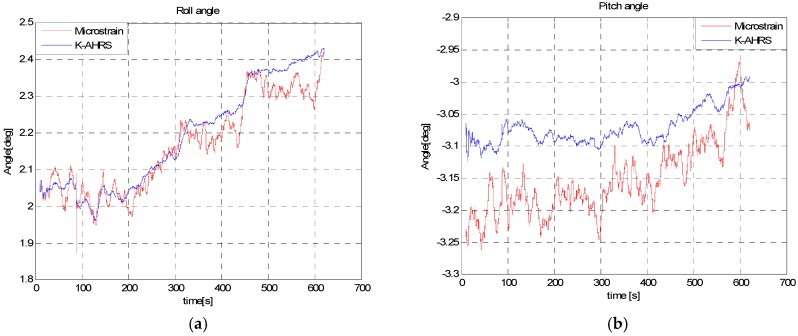
Roll angle (**a**) and pitch angle (**b**) estimate in static test with permanent magnet pass.

**Figure 20:**
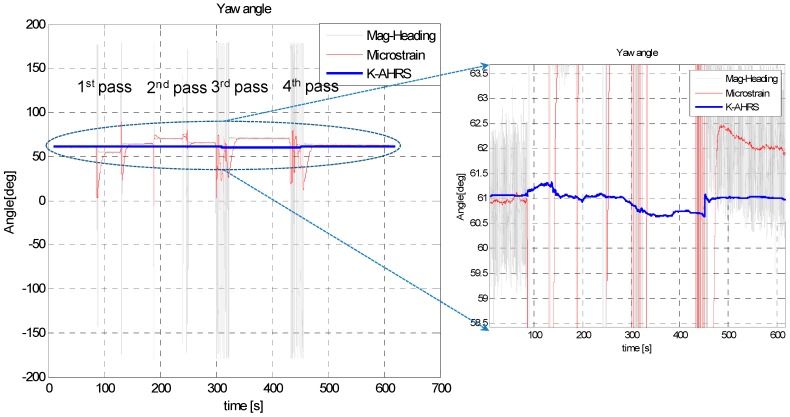
(**Left**) Heading angle estimate in the static test with permanent magnet pass; (**Right**) Zoomed-in view for visual inspection.

This test was conducted to check the performance of our proposed algorithm in a highly magnetic distortion environment. In this test, we kept the IMU stationary and passed a permanent magnet across the sensor back and forth, by approaching it from different directions, as shown in Figure 18. The permanent magnet bar was passed by four times close (<5 cm) to the IMU. The purpose of this test is to examine the effects of permanent magnet on the Earth’s magnetic field strength, dip-angle angle and, hence, on the resulting heading angle accuracy. The roll and pitch angle estimates are given in Figure 19 and the heading angle calculated from raw magnetometer data (tilt compensated), and the fused heading by EKF (called *K-AHRS*) is compared with reference AHRS (Microstrain’s) and initial heading angles, in Figure 20.

This experiment reveals the ability of our designed technique to reduce drift in attitude, even in the presence of a strong magnetic field. The *MAD* algorithm decides when to use the Earth’s magnetic field for heading angle calculation by checking the magnetometer data’s health. The maximum error in heading is <0.5 degree in this static.

### 7.2. Moving in a Straight Line Test

**Figure 21:**
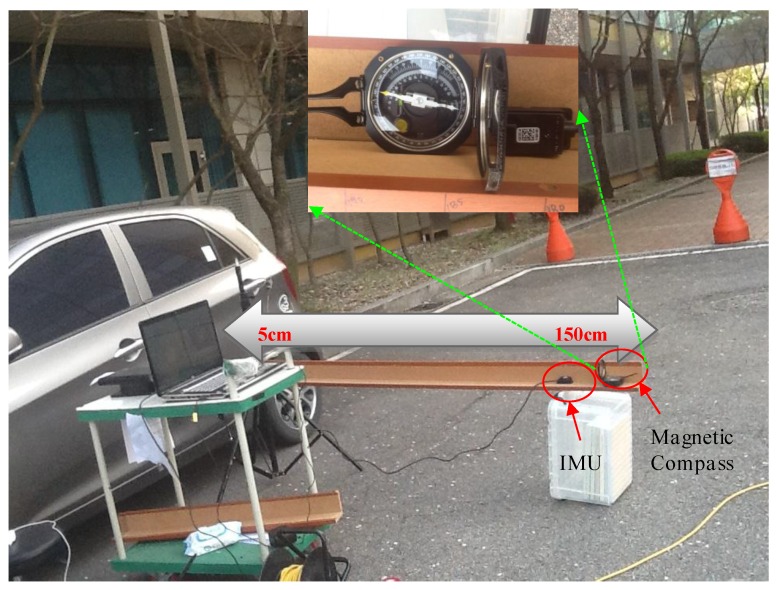
Moving IMU in an exact straight line, by sliding the sensor on a straight wooden support. In close-up is shown a very precise magnetic compass for reference heading.

In this test, we moved the IMU sensor along a straight wood support, making sure that the heading angle did not change during this motion. The sensor is moved close to a car (large metallic body) in intervals of 20 cm and then backwards, Figure 21. The aim of this test is to check the effect of magnetic interference on heading angle deterioration. The minimum distance to which the sensor moved towards the car is about 5 cm and then it was moved backwards on the same straight wooden support to its initial position (>150 cm). We examined the changes in the raw magnetic data, magnetic field strength, dip angle and heading angle corresponding to the distance from the car. The effectiveness of the proposed algorithm is compared with reference attitude (Microstrain AHRS) and constant initial heading angle.

**Figure 22:**
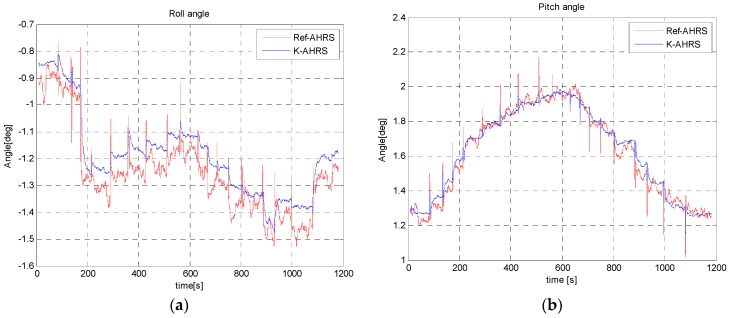
Roll angle (**a**) and pitch angle (**b**) estimation during dynamic test: moving the sensor towards a car as per experimental set-up in Figure 21.

In this in-motion test, we moved IMU towards a car (a large metallic object) from a distance of 150 cm to 5 cm on a straight wooden support. The roll and pitch angles are not effected by magnetic disturbances and follow the reference angles, as seen in Figure 22.

**Figure 23:**
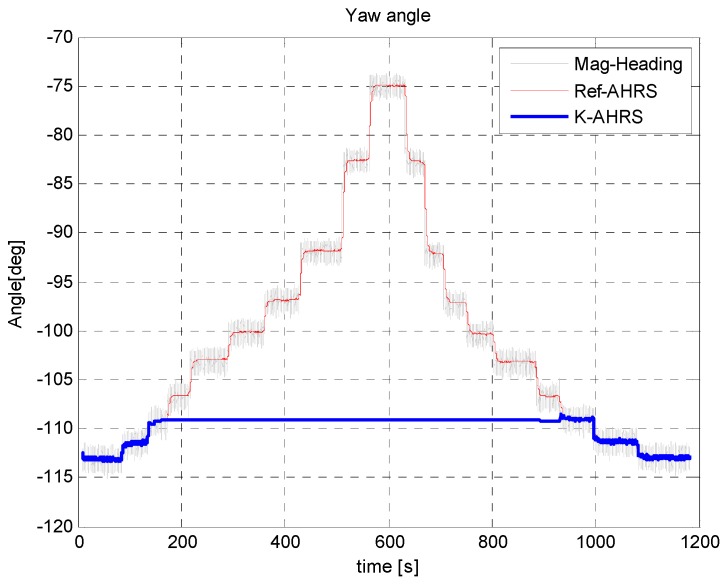
Heading angle estimate when moving a sensor towards a car. The maximum heading angle change is <3 degrees in highly magnetic distortion environment by applying the *MAD* algorithm.

**Figure 24:**
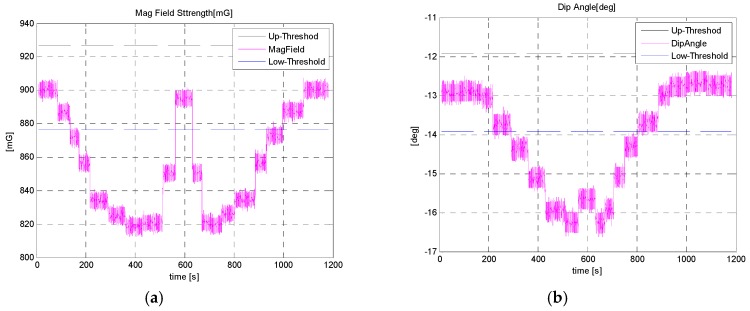
Magnetic field strength and dip-angle with thresholds applied for moving in a straight line test.

It is seen from Figure 23 below that as the sensor approached (<100 cm) the car, a large metallic body, the heading angle output of Microstrain AHRS and raw magnetic heading start deteriorating, whereas the heading angle estimated by applying our *MAD* algorithm is not effected much, even in the close vicinity (<5 cm) of the car. The corresponding magnetic field strength and dip angle applying the *MAD* algorithm are shown in Figure 24.

### 7.3. MAD Applied in a Dynamic Test in PNS Application

We applied *MAD* algorithm to our PNS application. In this test, the effect of magnetometer heading angle on the pedestrian navigation drift reduction is analyzed in a mixed indoor/outdoor environment test. This test is similar to the indoor test conducted previously in Section 6.3, except that this time the magnetometer data is also recorded and incorporated in the EKF framework for heading correction using the *MAD* algorithm. The user started his walk outdoors in a magnetically clean environment and then after some time entered the same building as in the previous indoor test. The user walked on the first and second floors of the building and then came out and stopped at the same initial starting position. The *MAD* algorithm is applied throughout this test to correct the cross-track position error. The *MAD* algorithm is applied only when the foot is in stance phase. Even indoors, there are occasions when the magnetometer can find a clean environment, hence, its data is used for heading correction at those instances when walking indoors.

**Figure 25:**
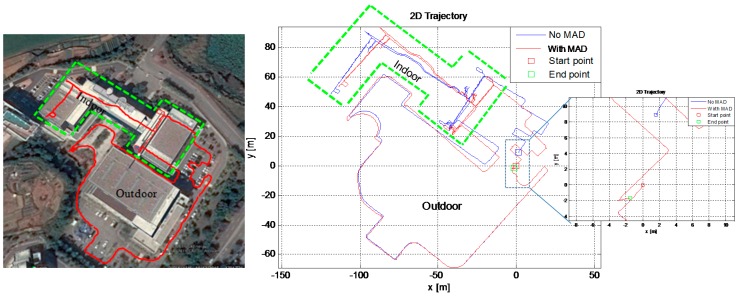
Effect of *MAD* algorithm on 2D trajectory estimation in PNS application, in a 30 min walk in a mixed outdoor/indoor environment. (**Left**) An approximate walking trajectory is drawn on Google Earth map; (**Right**) Estimated trajectory in PNS.

The ZUPT-only aided INS (*No MAD condition*) maintains an accurate trajectory estimation for some time, but as time goes on, the drift due to non-observability of heading angle increases. When aided with magnetic heading, after passing through the *MAD* algorithm, the drift in heading and hence the estimated trajectory cross-track error reduces drastically. It is seen in Figure 25 above that the *MAD*-applied trajectory (red) is more similar to the actual building layout on the left, because it stays parallel or perpendicular to the building layout, whereas the ZUPT-only aided INS trajectory (blue) diverges over time. The final position error is less than 4 m in 2D and percentage error is <1 m of the total distance travelled, as given in Table 6 above. These test results conform to our supposition that adding magnetometer data for heading drift reduction and, hence, getting more accurate position track estimates is a viable solution for PNS applications, even in indoor environments.

## 8. Conclusions

In this paper, we presented a pedestrian navigation system using a single foot-mounted IMU and exploiting motion constraints of the walker as *virtual sensors*, with no prior information about the test environment and the user itself. Simple, yet very effective algorithmic techniques were presented to reduce errors in the conventional ZUPT aided INS for navigation in infrastructure-free environments in PNS applications. After conducting experiments in indoor/outdoor navigationally harsh environments, we conclude that errors in the IMU only navigation solution are reduced drastically by exploiting only the motion constraints of the wearer and without using any other external navigation sensors or having any prior knowledge about the user or test environment. Based on experiments conducted in different environments—indoors/outdoors, an urban canyon and under a forest canopy—we conclude that the overall position errors, when compared with initial and final position at the same starting point, are <2% of the total distance (for >1 km) travelled by using a single foot-mounted IMU as the navigation sensor and exploiting motion constraints only. The use of a magnetometer to reduce heading drift is also analyzed and it is found that having the aid of a magnetometer heading is a very powerful tool to reduce overall position drift, especially cross-track position errors, if the health of the magnetometer data is analyzed first before utilizing it for heading calculations, as done using the *MAD* algorithm in this paper. The tests are conducted for normal gait only. Other types of pedestrian gait are not considered in this work, e.g., brisk walk or very slow walk, crawling, running at different speeds, etc., and may constitute a future research topic.

## Figures and Tables

**Table 1:** RMS error in position, if PA-locking technique is applied in a static test.

Method	2D RMS Error (m)	3D RMS Error (m)	Remarks
PA-Lock Not applied	0.043678	1.817	Time: 15 min
PA-Lock applied	0.00079767	0.20591	
Improvement	40 times	10 times	

**Table 2:** RMS error in attitude, if PA-locking technique is applied in a static test.

Method	Roll RMS Error (°)	Pitch RMS Error (°)	Yaw RMS Error (°)
PA-Lock Not applied	0.3450	0.0919	24.482
PA-Lock applied	0.234	0.056	1.3125
Improvement	47%	62%	15 times

**Table 3:** RMS error reduction by applying STH technique for indoor environment test.

Method	2D RMS Error (m)	3D RMS Error (m)	Remarks
STH Not applied	4.01	5.13	Time: 15 min Distance: 380 m Error/100 m: ≈0.8 m
STH applied	2.12	2.50	
Improvement	89%	100%	

**Table 4:** RMS error if STH and PA techniques are applied in an urban canyon environment.

Method	2D RMS Error (m)	3D RMS Error (m)	Remarks
**STH + PA Not applied**	26.584	29.543	Time: 45 min Distance: 1000 m Error/100 m: ≈1.5 m
**STH + PA applied**	14.427	19.797	
**Improvement**	84.27%	49.23%	

**Table 5:** RMS error comparison if STH and PA techniques are applied.

Method	2D RMS Error (m)	3D RMS Error (m)	Remarks
STH+PA Not applied	24.422	30.192	Time: 40 min Distance: 11,000 m Error/100 m: ≈1 m
STH+PA applied	8.0627	20.606	
Improvement	202.9%	46.52%	

**Table 6:** RMS error comparison when *MAD* algorithm is applied in a mixed indoor/outdoor environment test.

Method	2D RMS Error (m)	3D RMS Error (m)	Remarks
*No MAD* applied	9.8	12.84	Time: 30 min Distance: 500 m Error/100 m: ≈0.7 m
*With MAD*	3.24	9.21	
Improvement	2 times	39%	
